# A Potential Bioelectromagnetic Method to Slow Down the Progression and Prevent the Development of Ultimate Pulmonary Fibrosis by COVID-19

**DOI:** 10.3389/fimmu.2020.556335

**Published:** 2020-12-04

**Authors:** Syed Muzzammil Masaud, Oliver Szasz, A. Marcell Szasz, Huma Ejaz, Rana Attique Anwar, Andras Szasz

**Affiliations:** ^1^ OncoTreatments Pvt Ltd, Karachi, Pakistan; ^2^ Biotechnics Department, St. Istvan University, Godollo, Hungary; ^3^ Department of Internal Medicine and Oncology, Semmelweis University, Budapest, Hungary; ^4^ Department of Biochemistry, University of Turku, Turku, Finland; ^5^ Department of Oncology, Nishtar Medical College Multan, Multan, Pakistan

**Keywords:** SARS-CoV-21, rehabilitation, electric field, immune-effect, heat-shock protein, modulated electro-hyperthermia

## Abstract

**Introduction:**

Right now, we are facing a global pandemic caused by the coronavirus SARS-CoV-2 that causes the highly contagious human disease COVID-19. The number of COVID-19 cases is increasing at an alarming rate, more and more people suffer from it, and the death toll is on the rise since December 2019, when COVID-19 has presumably appeared. We need an urgent solution for the prevention, treatment, and recovery of the involved patients.

**Methods:**

Modulated electro-hyperthermia (mEHT) is known as an immuno-supportive therapy in oncology. Our proposal is to apply this method to prevent the progression of the disease after its identification, to provide treatment when necessary, and deliver rehabilitation to diminish the fibrotic—often fatal—consequences of the infection.

**Hypothesis:**

The effects of mEHT, which are proven for oncological applications, could be utilized for the inactivation of the virus or for treating the fibrotic consequences. The hypothesized mEHT effects, which could have a role in the antiviral treatment, it could be applied for viral-specific immune-activation and for anti-fibrotic treatments.

## Introduction

The present global pandemic is a disease named COVID-19. It is caused by a coronavirus (SARS-CoV-2), that belongs to the order of Nidovirales, the family of Coronaviridae, and the subfamily of Coronavirinae (1). There are nearly 30 recognized coronaviruses (CoVs) that infect humans, mammals, fowls, and other animals (2). It is the third known zoonotic CoV disease after SARS-CoV-1 and the Middle East respiratory syndrome (MERS). The genome sequence of SARS-CoV-2 did not bear a close relationship to any of the previously identified CoVs (3, 4). The epidemiology research shows the validity of the complexity (long-tail distribution): SARS-CoV-2 characterizes by the 80/20 law (5); which means 80% of transmissions happen by 20% of the infected individuals, “superspreaders” (6). The SSs are not only spreading intensively, but it seems like they have a high mutation-activity too. The large dataset of genome assemblies show how genomic diversity was evolved from one common accessor (7), but rapidly mutated, and four major SSs developed during the spreading of the pandemic, different ones in different continents (8). Importantly, genetic diversity studies show that the mutations in the viral RNA sequence allow the interaction of the virus with the cells of the host’s immune system associated with CD8 T-cell and CD4 T-cell responses (9).

The COVID-19 is more serious than other viral infections starting with an upper respiratory infection (URI) because SARS-CoV-2 has multiple “faces”; it is a “great imitator” (10). It can have simple symptoms like a common cold with a runny nose, but it can have much more severe symptoms, like loss of smell, fatigue, vomiting, diarrhea, abdominal pain, muscle aches, even whole-body symptoms as rashes or spots of redness. Severe organ damage is also possible (heart failure, kidney damage, liver damage, etc.), which usually happens as a complication after the COVID period of the disease.

In contrast to earlier CoV infections, the patient who has SARS‐CoV-2 may remain asymptomatic in the first stage, which may progress into severe pneumonia (11), dyspnea, renal insufficiency, and even death (12). This long period of incubation without symptoms accelerates the pandemic because the virus carrier person is unknown for an extended period. The long incubation period and mild URI symptoms do not alert the patient, and many times, when the disease becomes severe, it is already too late to avoid the damages. The highly uncontrolled spreading among the population increases the total cases of the global epidemic, and contrary to the mortality observed in the cases of SARS-CoV-1 or MERS-CoV, the total number of deaths is significantly higher (13).

A highly possible hypothesis (14) speculates that the COVID-19 pandemic is completely underdiagnosed, and in consequence, the infection spreads silently across the entire globe. The conditions could develop clusters of severe infections among endangered subjects and randomly promote only lately recognized spots of death cases. However, it is another possibility that a significant number of individuals develop immunity against the virus, and the current danger naturally falls into a level of seasonal flu. Due to the uncertain future of the development, implementing reasonable measures to gain control over the development of the disease is the primary goal. The network analysis for blocking the slink spread of the virus may bear immense potential from public health perspectives (15). The fatality rate is very different by countries, ranging between 1%-14%, highly depending on the age of the infected population and comorbidities (16). The severity of the disease depends of the dose of infection (14). About 80% of the confirmed cases are asymptomatic or mild, 13.8% are severe, with symptoms including pneumonia and shortness of breath, and 4.7% of the patients get in critical conditions (17). All three human CoVs’ induced pneumonia. Severe lung involvement can be formed by excessive and aberrant non-effective host immune responses; furthermore, multiorgan failure could be developed, and the situation can easily become lethal (18–20).

In addition to the medical issues, social and global economic challenges are also incredible. Heavy social load appears with the suddenly overloaded medical capacities, which cannot adequately suppress the further escalation of the disease and serve the hospitalized patients who fall back upon critical care and need special technical conditions.

## Medical Challenges

Medical questions are complex and depend on multiple personal and conditional factors. The central question that needs to be answered for a step toward successful clinical therapy is the precise understanding of the mechanism of the infection, considering its complexity determined by the interconnected physiological feedback mechanisms of human homeostatic regulation. The human organism has developed defensive mechanisms to prevent the propagation and proliferation of invading pathogens. One of the most ancient types of immune reactions is the elimination of bacterial pathogen-infected cells by apoptosis (21). In plants, pathogens trigger a hypersensitive response (HR), inducing systemic acquired resistance (SAR) (22) as a kind of apoptosis. In animals and humans, homeostatic dynamic processes control the activation of apoptosis in a highly controlled manner. In both animals and plants, apoptosis is promoted by producing pro-inflammatory molecules associated with tissue development and homeostasis (22, 23). Despite the differences, both types of apoptosis are associated with the induction of similar morphological features, including membrane blebbing, cytosolic fragmentation, nuclear condensation, and fragmentation as well as biochemical events such as the degradation of genomic DNA, proteolysis, and redistribution of membrane lipids (24, 25). These changes are primarily due to the activation of caspases, a family of cysteine proteases (26, 27). Moreover, infected host cells produce defensins, host defense peptides of innate immunity (28), and induce pro-inflammatory cytokines with an important role in the clearance of the invading pathogen.

Similar to patients with SARS-CoV and MERS-CoV, some patients with SARS-CoV-2 develop acute respiratory distress syndrome (ARDS) with characteristic pulmonary ground-glass opacity on computer tomography (CT) images. In most patients with advanced disease, the SARS-CoV-2 infection is also associated with a cytokine storm, which is characterized by increased plasma concentrations of interleukins 2, 7, and 10, granulocyte-colony stimulating factor, interferon-γ-inducible protein 10, monocyte chemoattractant protein 1, macrophage inflammatory protein 1α, and tumor necrosis factor α (18–20, 29, 30).

At present, the clinical manifestations and severity of the COVID-19 epidemic are similar to SARS-CoV-1 (31) in inducing cytokine storm. The SARS-CoV-2 shares 79.5% of the gene sequence with the SARS-CoV-1 (32). Similarly to SARS-CoV-1, SARS-CoV-2 invades the alveolar epithelial cells by binding to the human spike protein recognition, angiotensin-converting enzyme 2 (ACE2) receptor (33, 34). The investigation of cell-membrane trafficking could identify the biological mechanisms behind the SARS-CoV-2 infection. The inhibitor of predominant cellular receptor ACE2 is a vasoconstrictor, which protects against organ damages; thus, it is an effective treatment option in hypertension, diabetes, and cardiovascular disease. ACE2 inhibitor, therefore, has a protective effect on ARDS, in which respiratory failure has a rapid onset of widespread inflammation in the lungs. The current statistical data shows poor prognosis related to the male gender, elderly ages, and comorbidities like hypertension, diabetes, cardiovascular diseases, which are also connected to ACE2 (35).

The other central player of the invasion of viral infection to the host cell is the extracellular matrix metalloproteinase inducer CD147 protein on the cell surface (36), extending the action of infection promoter ACE2 receptor. The invasion of the virus to the host cell *via* CD147 is a route that is added to the ACE promotion. The activity of CD147 grows under hypoxic conditions (37), which is usually generated by the massive ATP use of the viral infection of the cell. Blocking the CD147 protein could be a useful strategy for the prevention or first-phase treatment of SARS-CoV-2 viral infection, suppressing the possibility of developing the disease to a severe phase. It is well-known that CD147 appears in most malignant processes (38) involved in vascular endothelial growth factor production, and it could promote the tumor cell invasion and metastasis. The CD147 protein appears like a hallmark of cancer with metabolic reprogramming. CD147 enhances glucose metabolism (39), and it contributes to the immunosuppression by inhibiting the p53-dependent signaling pathway (40).

An appropriate blocking of the ACE2 and CD147 silences the common cell-entries of viruses and could give a solid base for successful therapy. However, their involvement in many independent signal pathways could cause a serious imbalance of the physiological regulations. This “double-edged sword” phenomenon makes the treatment very complicated. The infection by SARS-CoV-2 CoV shows the same complex phenomenon as the self-organized living organism in general (41). It means that processes in the development of the diseases are stochastic (time-dependent probabilities of the events) have promoters and suppressors, and their balances decide the infection’s fate. The cumulative dose of viral exposure and the efficacy of the local innate immune response (natural IgA and IgM antibodies, mannose-binding lectin) form the most important balance in the first 10–15 days of the infection (42). When the virus is able to block the defending primary innate immunity, it could rapidly spread from the upper airways to the alveoli, replicating itself without local protection. This phenomenon causes pneumonia and induces high antigen concentration clinically. The delayed and strong adaptive immune response (high-affinity IgM and IgG antibodies) that follows causes unstoppable inflammation and generates cytokine storm, mostly requiring intensive care and being fatal in some cases (30, 31). The balance of physical activity also has an effect: a low or moderate physical activity could be helpful, but an extreme one could facilitate the spread of the virus. The same problem could arise in the case of oral breathing with hyperventilation during the incubation processes. The virus bypasses the immune barrier and determines the rapid development of the disease. This well-presented spatio-temporal harmony (balancing the viral-load, repetitive infection, the timing of the immune actions, concurrent effects of personal immune regulations, etc.) of the development of infection decides the fate of the patient (42). Consequently, understanding the complex process of the development of the infection is crucial for the medical attention, prevention, treatment, and rehabilitation of the patients. The COVID-19 disease has many unusual aspects compared to other respiratory viral infections: severe lymphopenia, causing a deficiency in immune regulatory processes; a cytokine storm with an extensive activation of cytokine secreting cells with innate and adaptive immune mechanisms, leading to acute respiratory distress syndrome and multiorgan failure (43). Laboratory evidence of clinical patients showed that a specific T-cell response against SARS-CoV-2 is important for recognizing and killing infected cells (44), and the measurement of these could inform the design of prophylactic vaccines and immunotherapy for the future.

The host immunity in COVID-19 patients with different severity of the illness was compared after patient admission (45). Several laboratory values, such as ferritin, lactate dehydrogenase, and D-dimer, were increased in the tests of patients in severe and extremely severe conditions. The absolute numbers of CD4+ T cells (helper T-cells), CD8+ T cells (killer T-cells), and B cells significantly decreased, and the NK-cells significantly increased with the escalation of infection, while their percentage proportion did not change so markedly. The ratio of CD4/CD8 cells did not change, while the activation markers of CD4+ and CD8+ T cells (HLA-DR and CD45RO) increased, and the co-stimulatory CD28 decreased by the aggravation of the disease. The percentage of natural regulatory (suppressor) T-cells (Tregs) was decreased in extremely severe patients. The percentage of interferon-gamma (IFN-γ), a soluble cytokine, which is critical for innate and adaptive immune reactions, is produced predominantly by NK-cells and promoting the development of CD8+ and CD4+ T cells, increased by the development of the illness. Cytokines IL-2R, IL-6, and IL-10 were all increased in extremely severe patients, while the activation of dendritic cells (DCs) and B cells was decreased in extremely severe stages of the patients. Interestingly, the age of the patients in severe and extremely severe stages of the disease had no significant influence. Another remarkable observation is that the CD4+ and CD8+ T cells are involved in the pathogenesis of an extremely severe viral infection (45).

The early pathological changes made by SARS-CoV-2 show viral interstitial pneumonia, and diffuse alveolar damage in the lung, as well as pulmonary edema, is also observed frequently (46). It suggests that for patients with early-stage mild SARS-CoV-2 infection, even after their condition improves and is discharged from the hospital, there is a potential—hidden—the risk of further progression to pulmonary fibrosis (47). Viral pneumonia often causes acute idiopathic pulmonary fibrosis (IPF), and the exact mechanism is not yet evident, often leading to irreversible restrictive lung function deterioration and death, practically *via* suffocation. In the cases of those who survive intensive care, these aberrant and excessive immune responses lead to long-term lung damage and fibrosis, resulting in functional disability and reduced quality of life (48, 49). According to a meta-analysis of 50 466 COVID-19 hospitalized patients, 14.8% of COVID-19 patients developed ARDS (50), which is lower than the observed 20% of SARS patients; ARDS survivors often had pulmonary fibrosis, and 36% and 30% of SARS patients develop IPF 3 and 6 months after infection (51). Therefore, patients overgone by SARS-CoV-2 infection have the potential to progress to IPF, irrespective of how severe the disease was.

A large number of COVID-19 patients require mechanical ventilation to maintain respiratory function. This forceful mechanical interaction could cause ventilation-related lung injury (52). Extensive ventilation could facilitate the direct penetration of the high concentration of SARS-CoV-2 in the lower airways, escaping the impact on the mucosae with neutralizing antibodies (42). The adverse effects of mechanical ventilation are mediated by the systemic release of local inflammatory cytokines and the cellular, molecular mechanisms involved in lung injury caused by mechanical stress. The lung damage caused by mechanical ventilation can become an additional acute lung injury (ALI) (53). These changes’ underlying mechanisms may be an epithelial-mesenchymal transition (EMT) and the release of pro-fibrotic mediators caused by cell stretching and mechanical ventilation (54). The aggravating ALI also could induce pulmonary fibrosis.

## Pathological Needs

The recent understanding and the basic messages from the available literature of the COVID-19 research show how to continue the systemic investigations:

1. Many details of the development of the disease are unclear, first of all, the role of the innate and adaptive immune actions through the development of the severity of the disease.
2. Post-disease immunity and rehabilitation need more research data.
3. The spatio-temporal order of the infection looks significant, but it is not entirely understood. The dose and the repeated reinfection of the virus and the time development of the stages of the symptoms have more to investigate.
4. The importance of the spatio-temporal order is clearly shown by the request of the strong immune possibility at the beginning of the infection. However, the opposite happens: it suppresses the immune reaction while avoiding the cytokine storm when the severity of the disease develops.

## The Hypothesis

Our hypothesis is that the treatment of SARS-CoV-2 infected subjects could be successfully supplemented with modulated electro-hyperthermia [mEHT (trade name oncothermia (55))]. Our proposal focuses on the electromagnetic impact, which combines the effects caused by heat and electric field, using their strong synergy (56). The temperature-dependent and non-temperature dependent factors are combined for optimal treatment (57). The mEHT uses the biophysical differences between the malignant and healthy cells to select them and induce apoptotic signals (58); and immune-mediated abscopal effect (59–61). The technical details (62) and clinical achievements (63) are published elsewhere. The method has a long, successful history; it is applied in oncology worldwide (64). The inhibitory effect of mEHT to COVID-19 is hypothesized due to its immune effects and the biophysically driven selection of cells that create apoptotic signal transduction in the infected cells.

The mEHT could be used for three intentions, encompassing the entire medical activities used in the epidemic. The method could be applied:

1. for the treatment of patients suffering from mild and severe symptoms;
2. for the convalescence, recovering period, when the individual is discharged from the hospital but needs care for rehabilitation.

**Figure 1:**
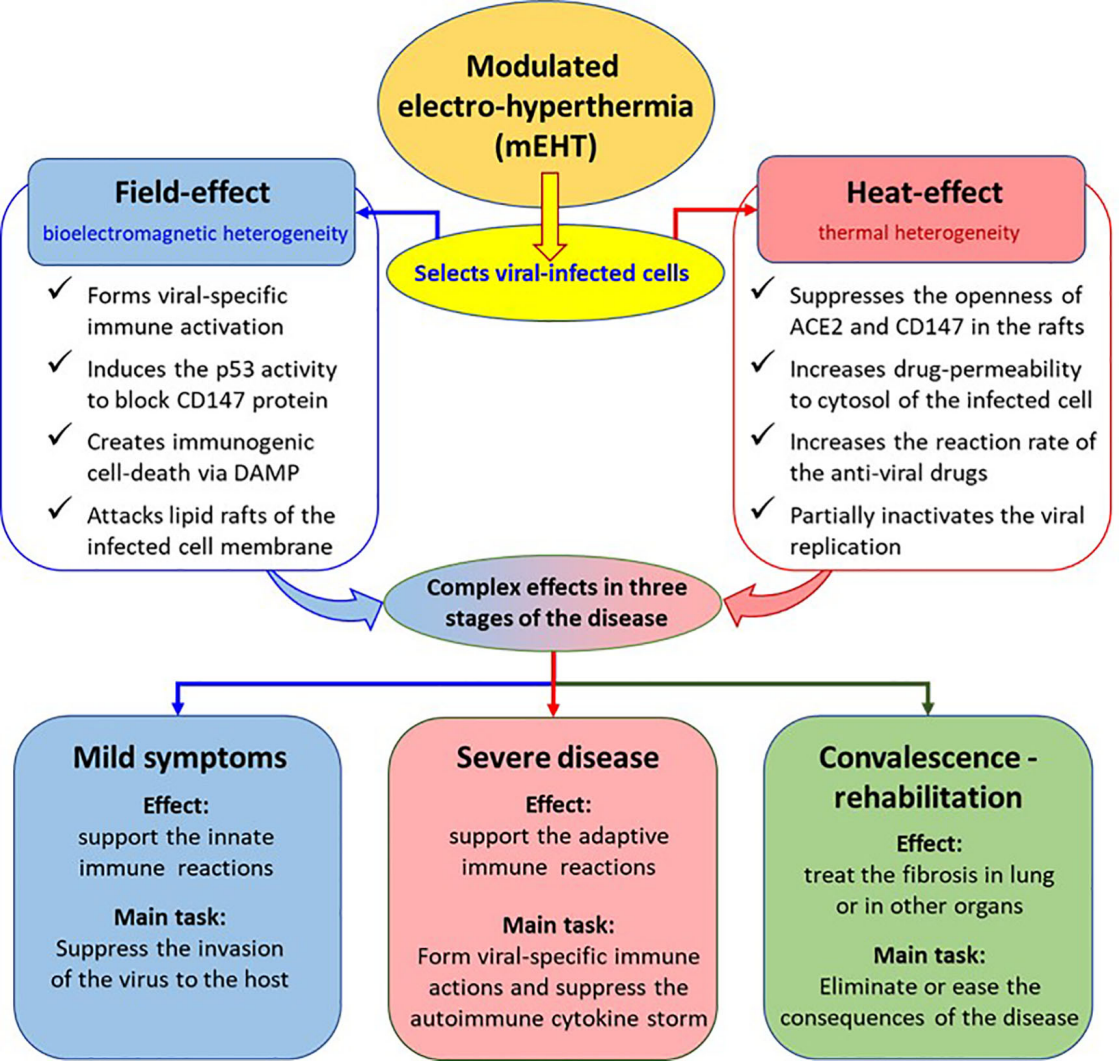
The possible action of mEHT treatment in COVID-19. The effects are deduced from results observed in oncology.

Our proposal is to use proper electromagnetic treatment, which may solve numerous challenges in SARS-CoV-2 viral infection and its consequences, based on the results achieved by mEHT applications that were proven successful in malignant diseases (63). We propose the application of a wide set of mEHT actions for the treatment of SARS-CoV-2 infection and its post-treatment syndrome (**Figure 1**).

## Selection of Infected Cells

**Figure 2:**
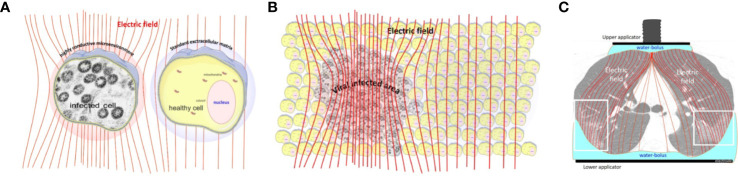
The radiofrequency current flows into the direction of the lower impedance. **(A)** The increased metabolic activity of the infected cells allow lower impedance (higher conductance) in their microenvironment than in the healthy cells. **(B)** The electric field selects the volumes with a high concentration of the infected cells. **(C)** the lung-lesions are loaded more with the radiofrequency current than the non-infected parts [The CT picture is from (74)].

SARS-CoV-2 infected cells have a higher metabolic rate, activating (65) and hijacking (66, 67) the energy from the host cell. The higher metabolic rate creates high ionic concentration in the near vicinity of the viral-infected cells. The high ionic concentration creates better conductivity in the cell’s microenvironment, which enhances the electric current in the area. This is the selection factor, which is extensively used by mEHT (68, 69). The selected cells will be the target of the electrothermal effect (70), which precisely attacks the targets only (71). The non-isothermal, certainly heterogenic heating process by mEHT selects the high conductivity cellular microenvironments (72). The high metabolic rate forms the change of the electric impedance. The enhanced metabolism causes high conductivity in the vicinity of the infected cells. Due to the intensive transfer of metabolites and other produced molecules, the concentration of the SARS-CoV-2 viruses is relatively high in the microvolume around the infected cells. Therefore, due to the relatively low electric impedance of the microenvironment of the infected cells, the mEHT selects them in the depth of the body like it does in the malignancy (73). The applied radiofrequency (RF) current drives the mEHT effect to the selected active infected cells (**Figure 2**). The treatment could break to the slower speed of the viral replication, which could be essential in processes when the adaptive immune defense is prepared. The slower replication activity could keep mild disease, which does not transfer to the severe stages.

**Figure 3:**
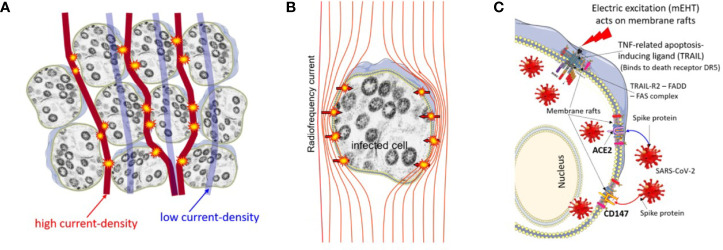
The high current density of the applied radiofrequency power excites the rafts at the cell membrane of the infected cells, which are selected by impedance differences. **(A)** The current flows dominantly in the extracellular matrix. **(B)** The current (energy absorption) excites the membrane rafts. **(C)** The ACE2, CD147, and DR5 death receptors are involved in the rafts.

The lipid rafts of the cell-membrane are involved in the actions of SARS-CoVs (75–78). A hypothesis had been published that presumes the active reduction of SARS-CoV-2 infectivity by lipid rafts, inhibiting the lipid-dependent binding to the host cells (79). Instead of the chemical reagents, mEHT could directly target the lipid rafts of the selected viral-infected cells, as it happens in a tumorous cell with higher metabolic activity (80) (**Figure 3**). The high energy absorption by selected rafts is supported well by in-silico models, too (81).

## The Effect Of Temperature

Unfortunately, the mitochondrial way of apoptosis in SARS-CoV-2 infected cells relates to the induction of viral pathogenesis, with a positive correlation between apoptosis and virus replication (82, 83). The observation of apoptosis in various SARS-CoV-2 infected tissues also suggested that the induction of caspase-dependent mitochondrial apoptosis could be vital for viral pathogenesis (84). The main action of apoptosis by mEHT uses an external apoptotic pathway, exciting the death receptors on the membrane surface (85); as well as some of the pathways are caspase-independent (86), which creates different apoptotic processes from the mitochondrial signal transfer, and could helpfully suppress the viral replication. The observation of Jun N-terminal kinases (JNK) as a dominant factor to induce apoptotic cell death in mEHT (87) supports the expectations of suppressing the viral replication process.

**Figure 4:**
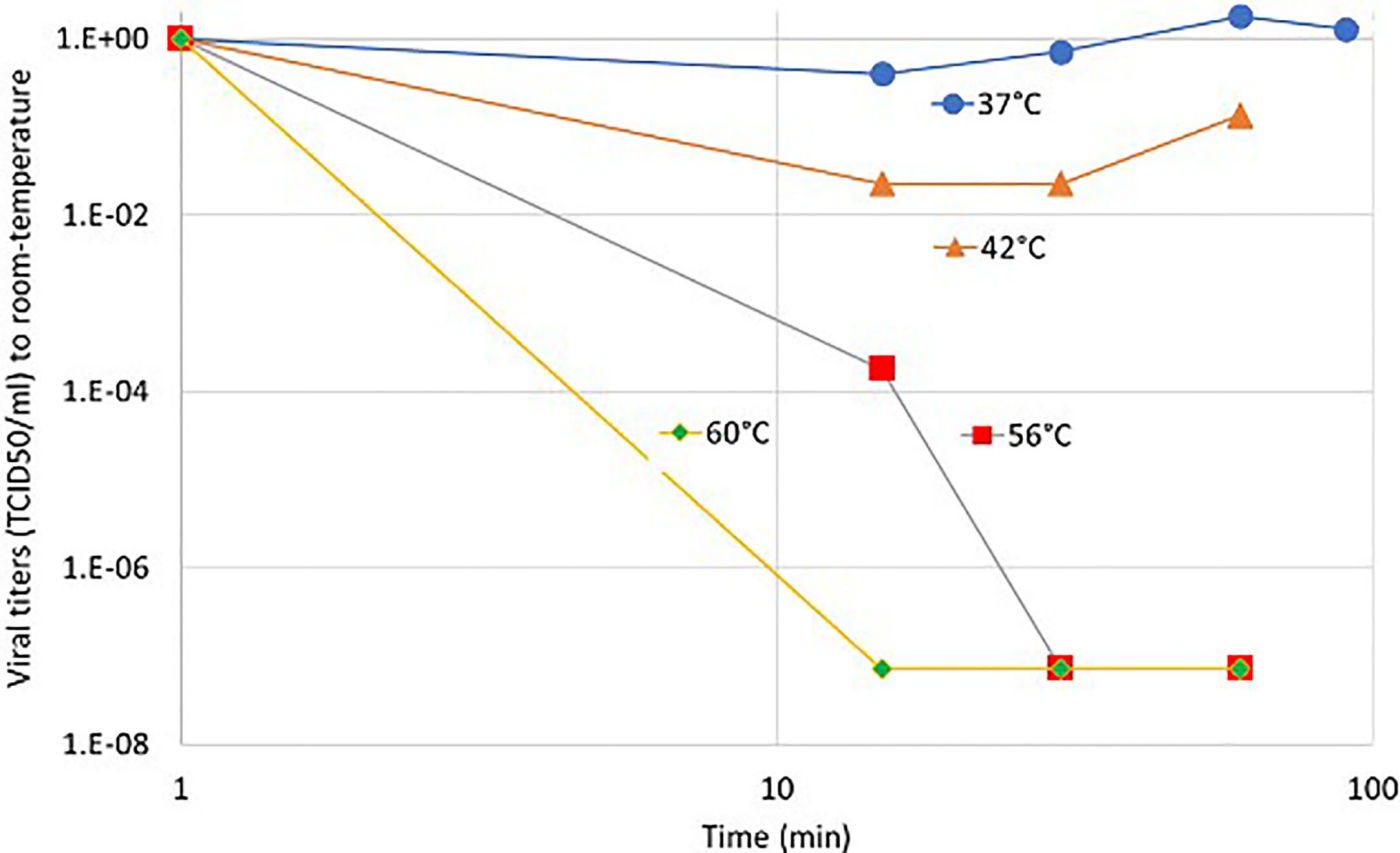
The relative variation of the viral titers (TCID50) of sputum by temperature compared to room-temperature.

Nobel-laurate A. Lwoff had shown in extensive researches that viruses are usually sensitive to temperature at a cellular level in living conditions. Studying the poliovirus, he had shown an optimal interval of the temperature where viruses are active, introducing infraoptimal and supraoptimal threshold temperatures when the yield of virus-replication decreases by 94% (88). For polioviruses, the infraoptimal (*t* ^+^) and supraoptimal (*t* ^-^) thresholds are: *t^+^* =38.5°C, *t* ^-^=30.5°C. He had shown no viron production at 39.5°C He also studied the pH dependence of viral reproduction, which shows a strong inactivation of the virus in the acidic environment (89). These observations were made much before the recognition of human CoVs. In the SARS-CoV-2 infection, the thermal aggregation of membrane proteins happens (90). In an earlier study (91), the temperature of the infected cells increased with higher metabolism. During the invasion of the host cell, the virus uses ACE2 and binding sialic acid on the cell membrane through hemagglutinin (HA) (92). The virus infection uses several metabolic systems in the host, using large amounts of energy, developing heat as well, which could increase the temperature of the host-cells by approximately 4°C–5°C, while the amount of available ATP decreases as soon as 3 h after the invasion (91). This intensive ATP consumption in a short time causes a sudden temperature increase. In light of the recent literature, the additional heating to the viral infection could create complications. The SARS-CoV-2 virus is stable at a higher temperature, while other coronaviruses are thermosensitive. Heating the body in a wet environment (like a sauna) could be the solution by the inhalation of air with a high temperature (93). The complete inactivation of the virus needs a higher temperature (94); with a phase-transition-like phenomenon at 56°C, which is well over the physiological limit of the body temperature. The viral load measurements in the sputum show the partial inactivation of the viral load at 42°C for 15 min (95) (**Figure 4**).

**Figure 5:**
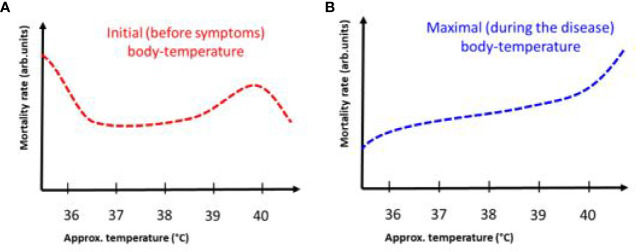
The dependence of mortality on body temperature before and during COVID-19 infection. The trend between the two measures is different. **(A)** Mortality rate as a function of maximum body temperature at the onset of symptoms. The 40ºC bulge is probably due to an independent inflammatory effect, increasing mortality at the end of the day. **(B)** The mortality rate versus the maximum body temperature during the disease has a permanent increase.

The partial inactivation is time-dependent and accompanied by the long-existing fever of the infected patient; it could promote other effects in the complex homeostatic regulation of the human body, while the electromagnetic interactions eliminate the load (96, 97) (**Figure 5**).

The thermal aggregation of the SARS-CoV membrane protein may be one of the reasons for the inactivation of this virus by heat (98). The mEHT is also a partly thermal method (99), which locally heats the membrane rafts of the targeted cells in-depth (100).

When cells are exposed to heat-shock, cells develop chaperone proteins (heat-shock proteins, HSPs) protecting the cells from the negative effects of the heat (101). In general, any stress of the cell develops these very conservative proteins as the most effective general protection against stresses. HSPs are involved in the repairing mechanisms of stress-induced damages, as well as participate in multiple signal-pathways, including many apoptotic signal transmissions. Several HSPs (denoted by their molecular weight in kD) are developed in the cell, involving different functions in the combat with the stress. The most important HSP homilies are the 60, 70, and 90 kD proteins (HSP60, HSP70, and HSP90). HSP chaperones have a wide range of applicabilities in medicine (102): thermal stress induces the most HSP70 (103); respiratory hyperthermia induces cytoprotective heat shock response in vesicular stomatitis virus (104) and rhinovirus-infected cells (105); the selective stress of mEHT method builds up HSP for supporting cell-resistance (106) and immune effects (107). HSP70 has an antiviral effect, so we expect that the heat and electric shock presented by mEHT induces a protective chaperone expression against the viral invasion in uninfected cells. The general antiviral effect of hyperthermia is also shown (108). HSP70 does essential information transfer for virus-specific immune action by antigen-presenting cells (APCs) (see below).

## The Immune Stimulative Effects

mEHT is an immune-stimulatory treatment (109–111). Immune effects are important factors against viral invasion (112). The desired effect may be induced by an appropriate HSP expression (113), like the immune action, it suppresses the human papillomavirus (114), and even human immune deficiency virus (115). We expect the immune-responses presented earlier in the case of other viruses also for patients suffering in COVID-19 (116). This expectation induces recommending the same therapy as well (117). Using the immunostimulatory effects of the autoimmune reactions (118) and fever (119) could be a helpful therapeutical strategy when it is controlled and does not allow an overreacted stormy feedback (120, 121). The clinical statistics of SARS-CoV-2 infected patients show that significantly more patients had fever among the cases where no critical care was necessary (122). A sign of the advantage of hyperthermia could also be that ACE2 inhibition was proven in preclinical experiments (123).

**Figure 6:**
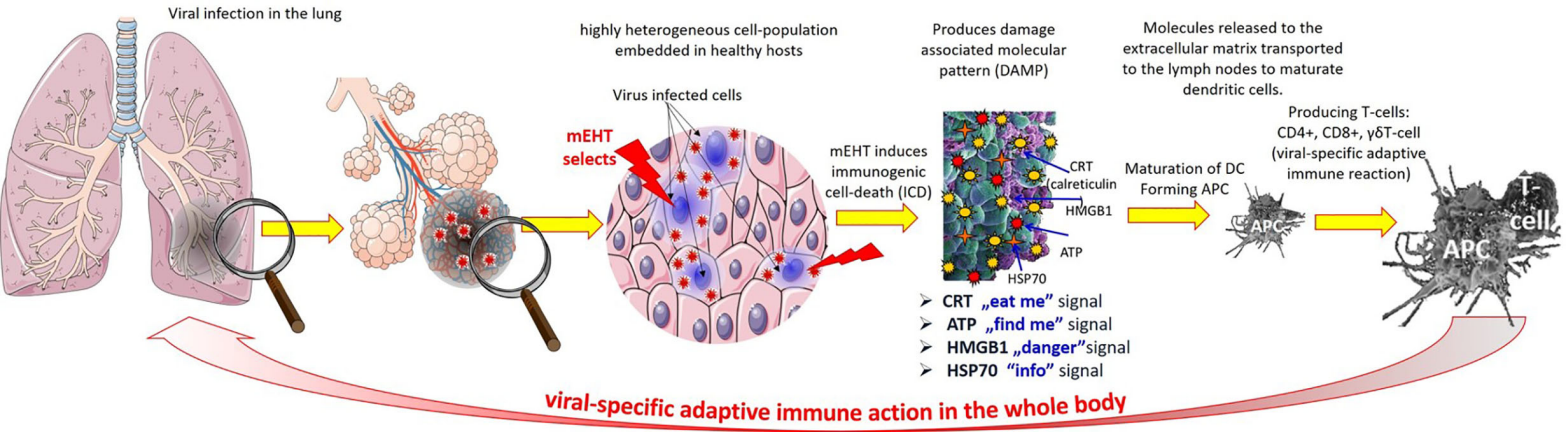
The antiviral immune-effect of mEHT: the mEHT selects the infected cells which release a damage-associated molecular pattern through the process of immunogenic cell-death. The DAMP provides viral genetic information to maturate the dendritic cells producing antigen presentation for T-cells, which turn to viral specific CD4+ CD8+ form. The viral-specific T-cells attack the infected cells in all over the body.

The mEHT effects could be divided into two categories: heat effects and field actions (124). Heating increases the intracellular pressure (70) and could damage the membrane, causing the direct necrosis of the cell, blocking the further replication of the virus by the nucleus. Importantly, the drug permeability to the cell is promoted by combined heat and field actions (125), which could be an important factor for the newly approved drug Remdesivir, which blocks RNA-dependent RNA polymerase (RdRp) in the nucleus (126). The field effects are mostly molecular and—in consequence of the molecular changes—immunological (127). The absorbed energy triggers extrinsic apoptotic signals, which are going through their pathway and could excite the variants of the apoptotic processes (107). A damage-associated molecular pattern (DAMP) is formed, which—when properly appears in time and localization—could complete the apoptosis as immunogenic cell death (ICD) (128). In the ICD process, the freed special DAMP molecules - like the 70 kDa heat-shock proteins (HSP70), calreticulin, HGMB1, and ATP—provide “info signal”, “eat me signal”, “danger signal”, and “find me signal”, respectively. These molecules provide infection-specific information for immune actions (129). The information carrier HSP in the extracellular electrolyte could serve as the surveillance information for the immune-system (130). The DAMP-induced ICD forms antibody presenting cells (APC) by the maturation of the dendritic (DC) producing immune actions by forming a virus-specific killer (CD8+) and helper (CD4+) T-cells directly (131). The immune-effects are well-proven in preclinical (132) and clinical (133) practice (**Figure 6**). The virus adapts to the immune system of its host (the human population) by natural selection, but the *in situ* produced genetic information to the APC production could compete with the adaption and stop, or at least suppress the viral replication.

The virus infects the immune cells due to its cross-immunity with common-cold immune reactions (9). The viral-load affects the interaction with the cellular immune response helper (CD4+) and killer (CD8+) T-cells in cooperation with the B-lymphocytes. These cells produce different cytokines: the helper T-cells produce interferons and interleukins, while the killer T-cells damage the viral-loaded cells by cytolysis and necrotizing cytokines. The starting immune reactions to SARS-CoV-2 have a competition between the infecting viral load and the innate immune system’s activity, especially the NK-cells, the already primed CD8+ cytotoxic T-cells and their activity and nonspecific or other CoV-related mucosal IgA levels with their T-cell interactions. An increased cytokine level [tumor necrosis factor-alpha (TNF-α) and interleukin-6 (IL-6)], as well as chemokines secreted in correlation with the migration of NK cells and macrophages in the virally infected area. A problem arises when the viral load dose is too high or the innate immune system is too weak. Due to the longer time to develop the adaptive immune system, the immune reply is not immediate. During the time of missing the complete adaptive reaction, the viral expansion could intensify, and the task for an adaptive immune system becomes more difficult. A second wave of the cytokine production starts [TNF-α, IL-6, gamma interferon (IFN-γ), IL-2, and IL-5], which may cause a cytokine storm, and could coincide with pneumonitis, developing a high risk of an extremely severe illness. Therefore, it is indicated (134) that leukocyte-mediated antiviral responses have a double-edge sword role that may contribute to the clearance of SARS-CoV but pneumonitis as well. The viral dose and the timing in the infective development have a decisional role in the actual conformation of the disease’s status and severity. The clinical intention is the prevention of the development of severe disease when the patient only has mild symptoms of the infection. An immunological way is a perfect option when the T and B cell immunity with virus-specific antibodies could inactivate or slow down the development of the diseases (43). The mild disease develops these virus-specific immune cells in the right phase of the infection when these could successfully fight against the viral load. However, when the immediate action of the innate immune system is not able to compensate the viral load, the developed cytokine storm and the viral load together becomes too extensive, and the adaptive immune reaction is late; therefore, the cytokine storm develops further, and the disease reaches a severe form.

The mEHT induces the p53 activity and causes apoptosis (125), which could arrest the accelerated viral load by arresting the invasion CD147 by the active p53. The present clinical studies with the CD147 blocking drug Meplazumab are feasible and can prevent SARS‐CoV‐2 spike binding and the subsequent infection (135). It may also have beneficial effects on other COVID‐19 treatments (136). The mEHT could be a significant addition as a complementary application with Meplazumab. Supporting the innate immunity and preparing the action of the adaptive answer at the appropriate time and force with mEHT could be crucial in blocking the infection from turning into a severe phase.

## Treatment of Fibrosis

Here we provided a theoretical basis for the utilization of modulated electro-hyperthermia against COVID-19 and other coronaviridae. The preventive effect is both primary (antiviral) and secondary (anti-fibrotic). In the long run, the latter will be responsible for decreased pulmonary capacity and eventual fatal outcome.

Therefore, in CoV pneumonia cases, it is important to control cytokine production and inflammatory response, given that they are responsible for the accumulation of cells and fluids. This strategy is challenging as we have not yet clearly identified any features in an immune response that can be inhibited specifically without compromising the beneficial host defense (12).

The mEHT is a good tool to treat fibrotic damages of patients during and after a viral infection and ALI, which releases mediators for the fibrosis (53). The EMT promotes the fibrotic processes (54, 137). The mEHT is a good tool to moderate the EMT by the electric field (138). Furthermore, HSP development is also a great possibility to reduce the effects of fibrosis, which is suppressed by the presence of these chaperones (139). The effect of HSP on fibrotic lesions is shown by the cure of warts too (140, 141). The high-dose vitamin C mEHT is inhibitory of the fibrosis in consequence of viral infection (142). The mEHT with low-frequency modulation on a high-frequency carrier (62) allows a deep penetration that reaches the infected cells in the lung. The demodulated signal promotes re-establishment of the homeostatic balance of the cells by charging the redistribution (143) and by the effective intracellular reordering of the cytoskeleton (144), giving less opportunity of the cytoskeleton components (structural: tubulin, actin; and dynamical: dynein, kinesin, and myosin) (145) providing intracellular transport to the correct location for the replication of the virus. Patients with penile fibrosis (Peyronie’s disease) (146), and palm fibrosis (Dupuytren’s contracture) (147) were successfully treated with mEHT. Furthermore, the mEHT treatment on malignant fibrosarcoma also showed a great benefit for the patient (148). The mEHT is successfully applied as a complementary treatment for lung cancer with high-dose vitamin C (149). Lung effusion is also one of the patient’s remaining negative states cured of the viral disease of SARS-CoV-2. mEHT offers a solution to this problem, too (150). Note that RF-current is widely used for cellulite fibrosis (151) and skin laxity (152), but only for near-surface areas. The mEHT is active in-depth (73), so the usual activity against the fibrotic structures is anyway expected.

The allometric fractal considerations help the solution of structural and metabolic problems of SARS viruses (153). The mEHT applies time-fractal modulation, which drives the processes to the direction of the healthy homeostatic balance, promoting the immune-system for surveillance (154). Mechanistic investigations of mEHT effects on related lung fibrotic animal models can be effectively conducted using *in vivo* molecular imaging outcome measures (155) and fractal dimension analysis of X-ray computed tomography (156). The mEHT acts on the fractal complexity (157). It uses an allometric approach (158) for self-similarity (41) of homeostatic systems (159), which could be an additional support to its action against forming IPF. The mEHT method, with its fractal-physiology considerations, could be a help to find the best treatment option. We are convinced that the disease’s complexity could be handled only by the complexity of the treatment. A miracle single curing effect is unrealistic.

The adverse effects of the mEHT in oncological applications are few, and it may reduce the side effects of the other therapies (160), and it has pain-reduction possibility too (161).

## Summary

**Figure 7:**
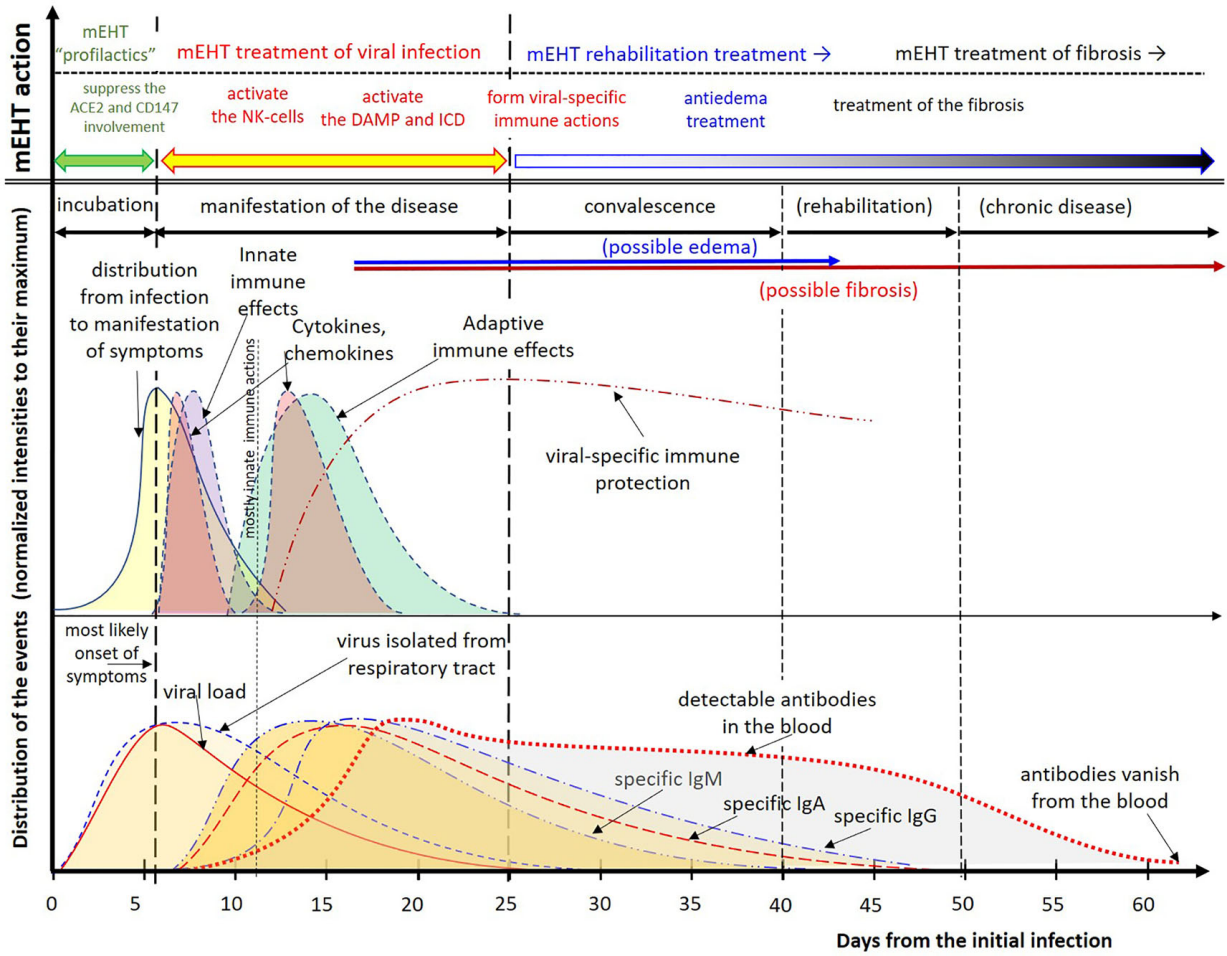
Summary of the viral effects of SARS-CoV-2 and the hypothesis based proposal for treatment. Due to the considerable individual differences, the distributions are normalized to their maximum. The intervals are approximate. The distributions are composed to their most likely form using the results (71, 107, 162–169). The disappearance of antibodies to SARS-CoV-2 was measured too (170), but others measured the opposite, existing antibody protection at least for two years (171).

The above hypothesis is comprehensively summarized in **Figure 7**. The distributions are not symmetrical (not normal distribution), having a longer “tail”. The tail is a typical fingerprint of the complexity (multiple feedback interactions with stochastic results) (172). The complexity depends on the patient and the disease’s general conditions, so the distribution curves are only orienting the reader; they could not be exact. The intensities of the effects also have individual dependencies, so all are normalized to their maximal values.

We conclude that the hypothesis that mEHT could be a helpful treatment in all phases of the COVID-19 has feasible references. Experimental and clinical data are mandatory and warranted.

## Data Availability Statement

The original contributions presented in the study are included in the article/supplementary material. Further inquiries can be directed to the corresponding author.

## Author Contributions

All named authors take responsibility for the integrity of the work as a whole and have given their approval for this version to be published. All authors contributed to the article and approved the submitted version.

## Funding

This work was not supported by a company, but it was supported by the Hungarian Ministry of Finance grant: PM/15237-13/2020.

## Conflict of Interest

SM was employed by the company OncoTreatments Pvt Ltd. AS declares that he is the Chief Scientific Officer of Oncotherm Kft./GmbH. OS declares that he is employed by Oncotherm Kft./GmbH.

The remaining authors declare that the research was conducted in the absence of any commercial or financial relationships that could be construed as a potential conflict of interest.
