# Corrigendum: A potential bioelectromagnetic method to slow down the progression and prevent the development of ultimate pulmonary fibrosis by COVID-19

**DOI:** 10.3389/fimmu.2022.1094086

**Published:** 2022-11-22

**Authors:** Syed Muzzammil Masaud, Oliver Szasz, A. Marcell Szasz, Huma Ejaz, Rana Attique Anwar, Andras Szasz

**Affiliations:** ^1^ OncoTreatments Pvt Ltd, Karachi, Pakistan; ^2^ Biotechnics Department, St. Istvan University, Godollo, Hungary; ^3^ Department of Internal Medicine and Oncology, Semmelweis University, Budapest, Hungary; ^4^ Department of Biochemistry, University of Turku, Turku, Finland; ^5^ Department of Oncology, Nishtar Medical College Multan, Multan, Pakistan

**Keywords:** SARS-CoV-21, rehabilitation, electric field, immune-effect, heat-shock protein, modulated electro-hyperthermia

In the published article, there was an error in the Conflict of Interest statement. “SM was employed by the company OncoTreatments Pvt Ltd. AS declares that he is the Chief Scientific Officer of Oncotherm Kft. The remaining authors declare that the research was conducted in the absence of any commercial or financial relationships that could be construed as a potential conflict of interest.” The correct Conflict of Interest appears below.

In the published article, there was an error in the Funding statement. Missing grant supporting this manuscript. The correct Funding statement appears below.

## Funding

This work was not supported by a company, but it was supported by the Hungarian Ministry of Finance grant: PM/15237-13/2020.

## Conflict of interest

SM was employed by the company OncoTreatments Pvt Ltd. AS declares that he is the Chief Scientific Officer of Oncotherm Kft./GmbH. OS declares that he is employed by Oncotherm Kft./GmbH.

The remaining authors declare that the research was conducted in the absence of any commercial or financial relationships that could be construed as a potential conflict of interest.

The authors apologize for these errors and state that this does not change the scientific conclusions of the article in any way. The original article has been updated.

## Publisher’s note

All claims expressed in this article are solely those of the authors and do not necessarily represent those of their affiliated organizations, or those of the publisher, the editors and the reviewers. Any product that may be evaluated in this article, or claim that may be made by its manufacturer, is not guaranteed or endorsed by the publisher.

